# Do ChatGPT and Other Artificial Intelligence Bots Have Applications in Health Policy-Making? Opportunities and Threats

**DOI:** 10.34172/ijhpm.2023.8131

**Published:** 2023-11-26

**Authors:** Plinio Morita, Shahabeddin Abhari, Jasleen Kaur

**Affiliations:** ^1^School of Public Health Sciences, University of Waterloo, Waterloo, ON, Canada; ^2^Department of Systems Design Engineering, University of Waterloo, Waterloo, ON, Canada; ^3^Research Institute for Aging, University of Waterloo, Waterloo, ON, Canada; ^4^Centre for Digital Therapeutics, Techna Institute, University Health Network, Toronto, ON, Canada; ^5^Institute of Health Policy, Management, and Evaluation, Dalla Lana School of Public Health, University of Toronto, Toronto, ON, Canada

## Introduction

 Health policy-making is the process of developing and implementing policies that address public health issues and improve health outcomes. It involves the identification of health problems, the development of policies and strategies to address those problems, and the allocation of resources to support those policies. Health policy-making also involves working with various stakeholders such as government agencies, healthcare providers, patient groups, and advocacy organizations to develop policies that are effective, equitable, and sustainable. The goal of health policy-making is to promote health and prevent disease by addressing the underlying social, economic, and environmental determinants of health.^1,2^ The health policy-making process in recent years has faced various challenges, which have made the process more complex and difficult. The COVID-19 pandemic has had a significant impact on health policy-making by highlighting existing inequalities in healthcare access and outcomes, leading to calls for more equitable healthcare policies.^3^ Furthermore, the aging population has increased demand for healthcare services, particularly for chronic conditions, placing a strain on healthcare resources.^1,4^ Rising healthcare costs, advances in technology, and mental health have also emerged as major challenges that require policy-makers to develop policies that address these issues. Health disparities continue to exist across different populations, particularly among racial and ethnic minorities, further necessitating policies that promote health equity. These challenges require policy-makers to be innovative, adaptable, and responsive to the changing healthcare landscape.^3-5^

 As we navigate the ever-evolving landscape of healthcare, it is crucial to explore innovative solutions that can enhance the policy-making process. The growth of artificial intelligence (AI) in recent years had a significant impact on the healthcare industry. AI has the potential to transform healthcare by improving patient outcomes, increasing efficiency, and reducing costs.^6^ Furthermore, AI has the potential to revolutionize health policy-making. It has several potential applications in health policy-making, including improving the efficiency of healthcare systems, identifying areas where resources are most needed, and predicting and preventing disease outbreaks. Also, AI can be used to analyze healthcare data and identify patterns that can inform policy decisions, such as identifying population health trends and predicting the impact of policy interventions. Additionally, AI can help policy-makers optimize resource allocation by identifying where resources are most needed and how they can be used most effectively. Furthermore, AI can be used to predict and prevent disease outbreaks by analyzing patterns in health data and identifying areas where outbreaks are most likely to occur. Subsequently, the applications of AI in health policy-making have the potential to improve healthcare issues.^7,8^ ChatGPT that introduced in 2022, is a large language model developed by OpenAI. It is a language model that uses deep learning techniques to generate human-like text responses based on the input it receives. AI involves developing computer systems that can perform tasks that normally require human intelligence, such as perception, reasoning, and decision-making.^9^ Based on ChatGPT taxonomy in healthcare care, we can classify the applications of ChatGPT in some categories such as triage, translation, medical research, clinical workflow, medical education, and consultation. Applications of ChatGPT in health policy-making are related to the consultation category.^10^ AI bots, such as ChatGPT and similar models, offer promising prospects for improving the efficiency and effectiveness of health policy development. ChatGPT is an example of how AI can be applied to natural language processing tasks, and it has been trained on a massive amount of data using deep learning algorithms, which enables it to understand and generate coherent responses to text inputs.^9^ By leveraging natural language processing capabilities, these bots have the potential to streamline communication, facilitate data analysis, and provide valuable insights to inform evidence-based policy-making.

 The objective of this viewpoint paper is to explore the applications of AI bots, particularly ChatGPT, and examine their potential contributions to health policy-making. To broadly explore and reach a comprehensive perspective, we conducted a literature review. This involved a thorough search in respected databases such as PubMed, Scopus, Embase, and Google Scholar, utilizing specific keywords (“ChatGPT” OR “Natural Language Processing”) AND (“Health Policies” OR “Health Policy” OR “Healthcare Policy” OR “National Health Policy” OR “Policy Making”) to refine our results. To ensure precision, our search was restricted to English-language papers published prior to April 10, 2023. Following the careful evaluation of identified articles, some relevant papers were selected for detailed analysis. Subsequently, a qualitative content analysis and research panel were employed to extract and categorize pertinent insights regarding ChatGPT’s potential applications, opportunities, and associated challenges in the realm of health policy-making. The outcomes of this study aim to contribute to future research and policy development within the field of health policy-making. By providing a comprehensive understanding of the potential impact of AI bots, policy-makers and stakeholders can make informed decisions regarding the integration of these technologies into their practices. Additionally, this paper aims to support ongoing efforts to improve public health outcomes through evidence-based policy-making, leading to more efficient and effective healthcare systems.

## Potential Applications of AI Bots Like ChatGPT in Health Policy-Making

**Figure 1:**
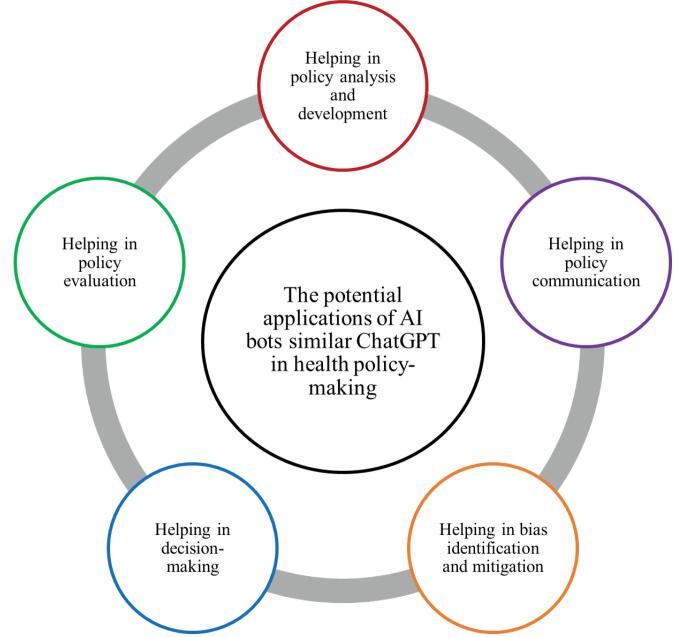
The Potential Applications of AI Bots Similar ChatGPT in Health Policy-Making. Abbreviation: AI, artificial Intelligence.

 At first, we identified the potential applications of AI bots like ChatGPT in health policy-making. Figure 1 shows these potential applications.

1. Policy analysis and development: AI bots, such as ChatGPT, can automate the process of policy analysis and development by generating policy recommendations based on data and evidence. This can save time and resources for policy-makers and help ensure that policies are evidence-based and data-driven. For example, ChatGPT could analyze global healthcare policies and research data to recommend optimal vaccination strategies during a pandemic, considering factors like disease spread patterns and vaccine distribution logistics.
2. Policy communication: Effective communication of policies is crucial for transparency and public engagement. ChatGPT can improve policy communication by generating summaries of complex policy documents in plain language for better understanding by stakeholders and the general public. This can improve transparency, facilitate understanding, and encourages greater engagement from stakeholders, resulting in a more inclusive and participatory policy-making process. For instance, healthcare policy-makers could deploy ChatGPT on their websites to engage with the public, answering queries and concerns about a new telemedicine policy, thereby fostering greater transparency and understanding.
3. Policy evaluation: The evaluation of policies is an important aspect of the policy-making cycle, as it provides insights into their effectiveness and identifies areas for improvement. AI bots like ChatGPT can enhance policy evaluation by analyzing and synthesizing large amounts of data to identify trends and patterns that can inform policy decisions. The synthesizing data here refers to ChatGPT’s capability to integrate information from different sources into a single, cohesive analysis, thereby offering a comprehensive view of policy effectiveness. These potential applications can help policy-makers identify areas where policies are working well and areas that may need improvement. As an example, policy-makers could utilize ChatGPT to analyze healthcare data to evaluate the success of a nationwide smoking cessation campaign, determining its impact on reducing smoking-related diseases.
4. Decision-making: Informed decision-making relies on access to real-time information and evidence. ChatGPT can support decision-making by providing real-time responses to questions and concerns from stakeholders and policy-makers. Through the different data processing techniques, these bots can identify trends, patterns, and correlations, enabling policy-makers to make informed decisions based on comprehensive analyses of policy outcomes. This can significantly affect evidence-based policy-making. For instance, during resource allocation planning, ChatGPT could analyze patient data to recommend the optimal distribution of medical supplies during a public health emergency, ensuring equitable access to critical resources.
5. Bias identification and mitigation: Ensuring fairness and equity in policy-making is of utmost importance. AI bots, such as, ChatGPT have the potential to help in identifying and mitigating bias in policy formulation and implementation. By examining the policy outcomes and comparing them across various demographic groups, these bots can flag inconsistencies or disparities. This helps policy-makers in addressing these issues proactively and designing policies that promote equity and inclusivity. For example, ChatGPT could be utilized to analyze healthcare policy documents and public health guidelines for potential biases related to gender, ethnicity, or socioeconomic status.^10-18^

It is important to emphasize that the cases presented in this viewpoint article signify potential applications of ChatGPT in health policy-making in the future and all of them are not currently specified applications in practice. As ChatGPT is a relatively new AI-based technology, substantiating its actual applications necessitates more rigorous and compelling evidence.

## Opportunities and Threats of Using ChatGPT and Other AI Bots in Health Policy-Making

**Figure 2:**
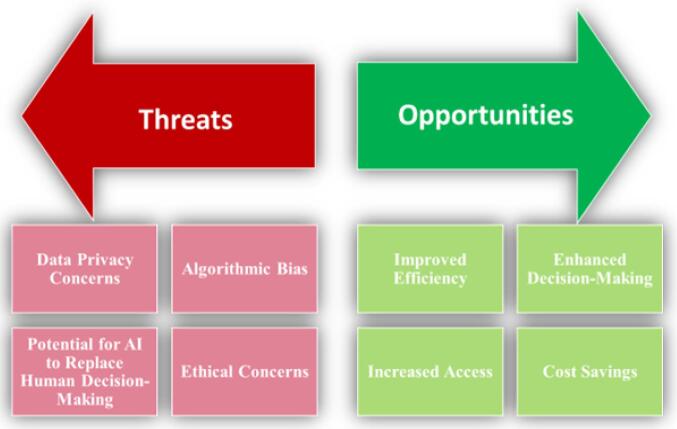
Opportunities and Threats Using ChatGPT and Other AI Bots in Health Policy-Making. Abbreviation: AI, artificial Intelligence.

 The application of ChatGPT and other AI bots in health policy-making presents a range of opportunities that have the potential to improve healthcare outcomes enhance decision-making processes. However, it is crucial to acknowledge and address the accompanying threats. This section will explore

 the opportunities^11,19-22^ and threats^4,11,13,21,23-25^ associated with the application of ChatGPT and other AI bots in health policy-making. Figure 2 shows these opportunities and threats.

## Opportunities

 Improved efficiency: A significant advantage of using AI bots for health policy-making is the ability to enhance the efficiency by automating tasks and expediting the data analysis. For example, by training ChatGPT on extensive healthcare datasets, it can identify patterns and trends, generate insights, and provide recommendations. This can help policy-makers in making well-informed decisions in less time, thereby reducing the time and resources required for manual data analysis and report generation.

 Enhanced decision-making: AI bots can also support decision-making processes by providing data-driven insights and recommendations. ChatGPT can be trained to analyze large amounts of data from patient health records, clinical trials, and other sources to identify trends and patterns in patient outcomes, disease prevalence, and other key indicators. These insights can be used to inform resource allocation, policy development, and other critical areas of healthcare decision-making.

 Increased access: AI bots can also be used to increase access to healthcare information and services by engaging with stakeholders, such as patients and healthcare providers, on a large scale and in a personalized manner. ChatGPT, for example, can be used to provide personalized health recommendations and advice to patients based on their health data and preferences. This expands access to healthcare information and services, particularly for individuals who may not have access to traditional healthcare providers.

 Cost savings: By automating certain tasks, such as data analysis and report generation, AI bots can help reduce the costs associated with health policy-making. ChatGPT can be used to generate reports on disease prevalence, patient outcomes, and other key indicators, thereby reducing the time and resources required for manual data analysis and report generation. As a result, healthcare organizations and policy-makers can achieve significant cost savings, allowing resources to be directed towards other critical areas of healthcare.

## Threats

 Data privacy concerns: One of the primary concerns associated with the use of AI bots in health policy-making is the potential for data privacy breaches. AI bots often necessitate access to extensive healthcare data, including patient health records and other sensitive information. If this data is not properly secured, it could be vulnerable to hacking and other cyber threats, thereby putting patients’ personal and confidential information at risk. Additionally, there is a risk that AI bots may inadvertently reveal sensitive information through their recommendations or analyses, potentially leading to discrimination or other negative outcomes.

 Algorithmic bias: Another potential threat linked to the use of AI bots is algorithmic bias. This occurs when AI algorithms are trained on biased or incomplete data, leading to biased recommendations or decisions. For example, if an AI bot is trained on data from predominantly white populations, it may not accurately reflect the healthcare needs and outcomes of other racial or ethnic groups. Consequently, this can lead to disparities in healthcare access and outcomes, thereby perpetuating existing inequalities.

 Potential for AI to replace human decision-making: There is also a concern that the use of AI bots in health policy-making could lead to a reduction in the role of human decision-making. While AI can provide data-driven insights and recommendations, it cannot replace the expertise and judgment of healthcare professionals and policy-makers. Additionally, the use of AI bots may lead to a lack of transparency in decision-making processes, as it can be difficult to understand the reasoning behind AI-generated recommendations.

 Ethical concerns: Ethical concerns are also associated with the use of AI bots in health policy-making. For example, if an AI bot is used to make decisions regarding resource allocation, there may be concerns regarding the fairness and transparency of these decisions. Additionally, there is a risk that AI bots may be used to replace healthcare professionals in certain roles, potentially leading to job loss and other negative outcomes.

 Furthermore, it is important to address additional challenges and concerns inherent to this subject. These encompass the potential risk of deskilling policy and healthcare professionals arising from the widespread utilization of AI bots, disparities in global access to ChatGPT and other AI bots, prerequisites for robust technical infrastructure, the necessity for comprehensive education for policy-makers, the current absence of well-defined regulatory frameworks, and notable issues like the lack of integration and fragmented health data repositories, coupled with the absence of comprehensive electronic health records systems.

## Conclusion

 In conclusion, the application of ChatGPT and other AI bots in health policy-making offers both opportunities and threats that requires careful consideration. We think AI bots have the potential to be applied for improving the efficiency and effectiveness of healthcare decision-making processes, by providing data-driven insights and recommendations. They can also help to reduce the workload of healthcare professionals, freeing up time for more complex tasks. But all these potential benefits should be studied practically in future investigations. On the other hand, there are several potential threats associated with the use of AI bots in health policy-making. These include data privacy concerns, algorithmic bias, the potential for AI to replace human decision-making, and ethical concerns.

 Healthcare policy-makers need to thoroughly evaluate these potential risks and challenges when making decisions about incorporation of AI into their policy-making processes. To maximize the benefits of AI in health policy-making and mitigate potential risks, healthcare policy-makers should prioritize ethical and responsible use of AI. This includes ensuring the proper security and protection of patient data and training AI algorithms on diverse and representative datasets to minimize the algorithmic bias.

 Furthermore, policy-makers should ensure that AI bots are used in collaboration with healthcare professionals and not as a replacement for human decision-making, to ensure that the expertise and judgment of healthcare professionals are properly utilized. In our viewpoint, the use of ChatGPT and other AI bots in health policy-making presents a opportunity to help improving healthcare outcomes and increase efficiency. However, it is important to approach the use of AI bots with caution and to carefully consider the potential threats and challenges associated with their use. By doing so, healthcare policy-makers can maximize the benefits of AI while mitigating potential risks, ensuring that the use of AI in health policy-making is both ethical and impactful.

## Ethical issues

 Not applicable.

## Competing interests

 Authors declare that they have no competing interests.
